# A Novel Machine Learning Framework for Comparison of Viral COVID-19–Related Sina Weibo and Twitter Posts: Workflow Development and Content Analysis

**DOI:** 10.2196/24889

**Published:** 2021-01-06

**Authors:** Shi Chen, Lina Zhou, Yunya Song, Qian Xu, Ping Wang, Kanlun Wang, Yaorong Ge, Daniel Janies

**Affiliations:** 1 Department of Public Health Sciences University of North Carolina at Charlotte Charlotte, NC United States; 2 School of Data Science University of North Carolina at Charlotte Charlotte, NC United States; 3 School of Business University of North Carolina at Charlotte Charlotte, NC United States; 4 Department of Journalism Hong Kong Baptist University Hong Kong Hong Kong; 5 School of Communications Elon University Elon, NC United States; 6 Department of Medical Informatics School of Public Health Jilin University Jilin China; 7 Department of Software and Information System University of North Carolina at Charlotte Charlotte, NC United States; 8 Department of Bioinformatics and Genomics University of North Carolina at Charlotte Charlotte, NC United States

**Keywords:** COVID-19, Twitter, Sina Weibo, content feature extraction, cross-cultural comparison, machine learning, social media, infodemiology, infoveillance, content analysis, workflow, communication, framework

## Abstract

**Background:**

Social media plays a critical role in health communications, especially during global health emergencies such as the current COVID-19 pandemic. However, there is a lack of a universal analytical framework to extract, quantify, and compare content features in public discourse of emerging health issues on different social media platforms across a broad sociocultural spectrum.

**Objective:**

We aimed to develop a novel and universal content feature extraction and analytical framework and contrast how content features differ with sociocultural background in discussions of the emerging COVID-19 global health crisis on major social media platforms.

**Methods:**

We sampled the 1000 most shared viral Twitter and Sina Weibo posts regarding COVID-19, developed a comprehensive coding scheme to identify 77 potential features across six major categories (eg, clinical and epidemiological, countermeasures, politics and policy, responses), quantified feature values (0 or 1, indicating whether or not the content feature is mentioned in the post) in each viral post across social media platforms, and performed subsequent comparative analyses. Machine learning dimension reduction and clustering analysis were then applied to harness the power of social media data and provide more unbiased characterization of web-based health communications.

**Results:**

There were substantially different distributions, prevalence, and associations of content features in public discourse about the COVID-19 pandemic on the two social media platforms. Weibo users were more likely to focus on the disease itself and health aspects, while Twitter users engaged more about policy, politics, and other societal issues.

**Conclusions:**

We extracted a rich set of content features from social media data to accurately characterize public discourse related to COVID-19 in different sociocultural backgrounds. In addition, this universal framework can be adopted to analyze social media discussions of other emerging health issues beyond the COVID-19 pandemic.

## Introduction

Social media platforms are important communication channels for public engagement of various health issues [1-4]. Through social media, the public can not only receive information from health agencies and news outlets about various health issues [5] but also actively participate in web-based discussions with peers and influencers to exchange opinions about these issues [6]. Social media platforms have been adopted in various health campaigns by both health agencies and concerned groups, including promotion of vaccination [7,8], exercise and healthy lifestyles, and smoking cessation [9].

During health emergencies, especially global infectious disease pandemics, social media has been used substantially by both individuals and organizations. Social media platforms were frequently used during previous public health emergencies of international concern (PHEICs), such as the 2014 Ebola outbreak [10,11] and the 2016 Zika pandemic [12,13]. Social media has also been intensively used during the current COVID-19 pandemic; *COVID-19* is currently the most mentioned keyword across all major social media platforms worldwide. Therefore, social media can be used for infodemiology studies [14-17] to better understand public concerns and make informed decisions regarding the COVID-19 pandemic as well.

Health emergencies are seldom an isolated health or medical issue. Pandemics, including the current COVID-19 pandemic, are almost always intermingled with complicated interactions of underlying societal and cultural factors that vary within and among countries. Consequently, discussions of these pandemics on social media include content not restricted to health, as observed during the 2014 Ebola and 2016 Zika epidemics [10,18-25]. During the current COVID-19 pandemic, it has also been demonstrated that various social and political issues are associated with the pandemic, including different views on nonpharmaceutical interventions (NPIs) such as mask-wearing, social distancing, and stay-at-home-orders [26-29].

To extract and analyze various content features in social media posts, natural language processing (NLP) methods such as linguistic inquiry and word count (LIWC) are usually applied [30]. However, although LIWC can cover a broad spectrum of topic features, it was not specifically designed for health-related topics. LIWC places more emphasis on psychological processes [31,32]. In addition, LIWC was developed almost exclusively in the Western sociocultural context and may not work well when analyzing discussions outside Western societies. During the COVID-19 pandemic, many discussions have been taking place on social media platforms in non–English-speaking regions, such as the Sina Weibo platform in China [33]. Alternative data-driven computational linguistic/NLP algorithms aim to deliver more natural insights directly from data, bypass various human assumptions, overcome lack of inclusiveness of features, and reduce potential bias [34]. Examples of commonly used techniques include word embedding, such as word2vec and doc2vec [35]. However, completely data-driven techniques can result in a lack of interpretability. For instance, the exact meanings of vectors resulting from the doc2vec algorithm are unclear, and it is usually used for classification purposes [25]. Similar to LIWC, it is still challenging to use the Chinese language as an input into these data-driven algorithms without extensive data preprocessing, which may result in a loss of subtlety of the content of the original Chinese post.

Because of these technical challenges, especially the lack of universally designed content analysis and feature extraction analytical workflow, few studies have compared social media discussion across different socio-cultural backgrounds with regard to the COVID-19 pandemic [36-38]. Cross-platform and cross-culture studies are infrequent and generally observational [39,40].

Therefore, we suggest that there is an emergent need to develop a more interpretable and universal content analytical workflow across a wide sociocultural spectrum during the current COVID-19 pandemic and future pandemics. Developing this analytical workflow will vastly expand our fine-grained understanding and characterization of the content features of discussions on health issues worldwide. Until such a workflow is achieved, we will not be able to effectively compare and contrast health communication patterns on different social media platforms worldwide. As such, we propose the following two major objectives in this study:

1. Develop a content feature extraction and coding scheme to characterize discussions about the current COVID-19 pandemic on major social media platforms across socio-cultural backgrounds (Twitter and Sina Weibo);
2. Compare and contrast content features of the most shared viral social media posts on Twitter and Sina Weibo through a comprehensive analytical workflow with state-of-the-art machine learning techniques.

## Methods

### Retrieval of Social Media Posts

We acquired social media posts on both Sina Weibo (colloquially referred to as Weibo hereafter) and Twitter from January 6 to April 15, 2020, for a total of 100 days. The reasons we used the same sampling period for the two social media platforms were as follows. 1) It made the sampling process consistent and directly comparable; 2) this study focused more on sociocultural than specific geospatial locations. Weibo is almost exclusively used by Chinese users, while Twitter users cover a much wider range of geospatial regions. Given the very different sizes and patterns of the epidemic in different countries, we suggested that having a consistent sampling period could reduce confounding factors such as actual outbreak size and its influence on public perception of COVID-19.

The Weibo posts were acquired via the application programming interface (API) of Hong Kong Baptist University in Python. We downloaded all Weibo posts during the sampling period without further sampling. There were around 4 million Weibo posts acquired and archived.

The tweets were acquired directly from Twitter via a contract between the School of Data Science, the University of North Carolina at Charlotte, and Twitter. The tweets were not retrieved by the commonly used Twitter API or other commercial APIs. The tweets were a 1% sample; however, given the adequately large sample size (more than 10 million tweets), we believe that this sample is a good representation of public discourse regarding the ongoing pandemic on Twitter.

The keywords used to retrieve social media posts were *COVID19*, *nCOV19*, *SARSCoV2*, their variants (*novel pneumonia*, *SARS*, *SARS2*, *COVID*, *coronavirus*), and other related medical/health terms (*GGO*, *PHEIC*, *pandemic*). Inappropriate, derogative, and discriminating terms such as *WuhanVirus*, *WuhanPenumonia*, and *ChinaVirus* were also included to increase the sample size for research purposes. Both original posts and reposts were retrieved if they included the search terms.

### Identification of Viral Posts

“Viral” posts were defined as those with large numbers of shares (also known as “reposts,” “retweets,” etc) on different social media platforms. There are other ways to define viral posts, such as number of comments or number of likes. However, comments may not align with the content and intention of the original posts, while liking would not necessarily propagate the original post on social media. Sharing through reposting or retweeting indicates that the user acknowledged the value of the original post and actively participated in its dissemination on social media. Therefore, the number of shares was used to define viral posts.

Nevertheless, the three types of potential definitions of “viral” post were highly correlated (Pearson correlation coefficient ρ>0.8 for each pair of viral post definitions). For example, it was very common for a highly shared COVID-19 post to receive many likes and comments as well. Therefore, we suggest that focusing on one definition of “viral” post was able to provide sufficient insights for the other two definitions.

To avoid oversampling during certain days when a cluster of viral posts occurred (ie, numerous posts occurred on the same day), we identified and selected the 5 most shared posts on Weibo and the 5 most shared posts on twitter throughout the sampling period. Practically, we ordered the posts by original posting date first and then ranked them based on the number of shares they received on each day. Due to the fast pace of social media, most viral posts received a majority of their reposts within a short period of time, and the overall lifespan of a viral post usually lasted less than 48 hours [25]. Eventually, a total of 1000 viral COVID-19 social media posts were selected, 500 for Weibo and 500 for Twitter. Because of the relatively large sample size and size of content feature set (discussed next), we believe the sample size is adequate to provide accurate, granular characterization of viral social media posts regarding COVID-19.

### Extraction, Annotation, and Quantification of Content Features

In this study, we developed a relatively novel and comprehensive content analysis workflow to characterize and quantify various content features of health-related social media posts. The creation of content features went through two rounds of iteration. In the first round, we used an open-coding approach to identify an initial set of features by manually analyzing a set of 200 randomly selected social media posts. Then, we randomly selected another set of 800 posts, combined them with the 200 posts, split them into 5 subsets (200 each), and asked five student coders to analyze them independently. The student coders were provided with the list of initial features and were all bilingual, with fluency in both Chinese and English. Moreover, the coders were asked to create new features if they were missing from the existing list. Finally, we refined the list of features based on our review, comparison, and evaluation of the coding results. A few new content features were discussed and added in this round. In the second round, we leveraged the refined features in screening, evaluating, characterizing, and validating a test set of 50 randomly selected posts by our research team. Note that posts in this set were not necessarily viral posts. As discussed later in this paper, randomly selecting posts increases the coverage of various topic contents in the posts. We performed several iterations of intercoder reliability analysis, discussions, and refinements to ensure clarity and consistency in the definition and coding scheme of the features. The intercoder reliability (kappa value) threshold was set as 0.8 before deploying more comprehensive coding. The coding scheme can be described concisely as follows.

Each feature was 0-1 binary coded (ie, a post either had or did not have the specific content feature). This coding scheme is more objective and easier to interpret than LIWC because it only considers the presence of a specific content feature. In addition, because of the objectivity of the coding scheme, there is no need to translate the social media posts, as the subtlety in the original post may be lost during the translation process.

The final version included a total of 77 content features that were grouped into 6 major categories, each major category with more specific features. The six major categories included clinical and epidemiological features (eg, mentioning any symptoms or signs, transmission, or diagnosis and testing); countermeasures and COVID-19–related resources (eg, mentioning face masks, other medical supplies, or disinfection); policies and politics (eg, mentioning social distancing or stay-at-home-orders); public reactions and societal impact (eg, preparedness, remote working, or college education); spatial scales (eg, local, state/provincial, national, or international); and social issues (eg, discrimination against certain countries, violence, uncivil language). Note that these content features were not mutually exclusive, and a post could have multiple features under the same or different major categories at the same time as long as the post contained the specific contents. For example, a single post could mention symptoms, diagnosis, risk factors, and clinical consequences. In addition, these content features were universally developed and objective; therefore, they could be applied in different sociocultural backgrounds without the need of translation, which is required in LIWC. The complete descriptions of these major categories and further specific contents within each major category are provided in Multimedia Appendix 1.

After the comprehensive coding scheme was established and the list of 77 content features was defined, we then coded the 1000 posts according to the coding scheme. For each post, the output was a 77-element 0-1 binary vector. A 1 indicated that the post mentioned the corresponding content feature, while a 0 indicated that the specific post did not mention that content feature. In general, the more 1’s (and hence, the fewer 0’s), the more diverse the topics contained in the post. Fewer 1’s indicated more focused topics in the post. The final output for the analytical workflow was a 1000 × 77 binary matrix that could be further divided into two 500 × 77 binary matrices representing viral Twitter and Sina Weibo groups, respectively.

### Descriptive Analysis of Viral COVID-19 Posts Across Social Media Platforms

We applied descriptive analysis to quantify and contrast the prevalence of content features in the most viral COVID-19 posts across the social media platforms Weibo and Twitter. The prevalence was defined as the percentage of number of 1’s across all the sampled posts in each content feature. Prevalence was bounded between 0 (ie, none of the sampled posts mentioned the content feature) and 1 (ie, all posts mentioned the content feature). A larger prevalence indicated that the corresponding content feature was more frequently mentioned in the viral social media posts regarding COVID-19.

We further applied a two-sample *z* test to investigate whether there was statistically significant differences in the two prevalence measures of the same content feature between Weibo and Twitter. Because the data were 0-1 binary instead of continuous, the *z* test was more appropriate than the *t* test or Kolmogorov-Smirnov test. The content features that had the most distinct prevalence measures between the two social media platforms were identified based on the *z* test.

In addition to comparing different social media posts, we also studied the associations between content features on different social media platforms. Pairwise Pearson correlation was calculated between each pair of content features in both Twitter and Sina Weibo posts. Pairs with statistically significant associations (*P*<.05) were identified. These analyses provide a comprehensive characterization on how viral COVID-19 content features are distributed and correlated differently on the two major social media platforms.

### Unsupervised Learning of Viral COVID-19 Posts Across Social Media Platforms

To further investigate the distributions and relationships among multiple content features simultaneously, we applied the *t* distribution stochastic neighbor embedding (*t*-SNE) technique. *t*-SNE is a machine learning dimension reduction algorithm. In contrast to the more commonly used principal component analysis technique, *t*-SNE can handle data that are not normally distributed, as presented in this study (ie, binary data) and is also commonly used in other studies involving large and heterogeneous data (eg, bioinformatics data [41]). Performing *t*-SNE provides a clear visualization of associations among content features in 2D space instead of the original complex 77-dimensional feature space.

*t*-SNE dimension reduction paved the way for subsequent clustering analysis. In this study, we applied unsupervised machine learning k-means clustering [42]. Note that we created 6 major categories of content features for our own manual content coding effort. These 6 categories were based on our observation and discussion about the COVID-19 pandemic and public discourse on social media. Data-driven clustering analysis (also known as unsupervised learning), on the other hand, enables the data to “speak for themselves” (hence, “unsupervised”). Data-driven clustering provides a new angle of identifying possible aggregations of content features. For example, frequently concurrent content features may not necessarily be clustered under the same major manually created categories. *k*-means clustering does not require a priori information from researchers on how the features should be grouped; therefore, it reduces potential bias. The optimal *k* value to perform *k*-means clustering was determined by computing and inspecting the total within sum of squares (TWSS) with a wide range of *k* values from 1 to 20. Although larger *k* values are usually associated with smaller TWSSs, they increase the difficulty of interpreting the clusters. We examined and contrasted the clustering patterns of content features in the most viral COVID-19 posts on Twitter and Weibo.

**Figure 1:**
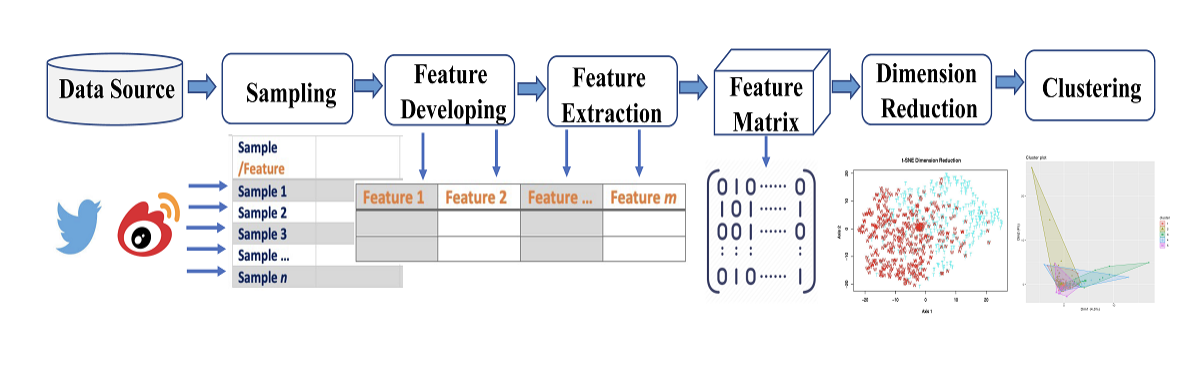
Conceptualized social media content feature extraction and analysis workflow. Sampling was performed with specific application programming interfaces in Python. Feature developing, extraction, and quantification were performed manually by our group. Subsequent analyses were performed in R.

The complete workflow of extracting and analyzing viral COVID-19 posts on different social media platforms is conceptualized and presented in Figure 1. All analytical codes were developed in R 4.0.2 (R Project) with supporting packages of *Rtsne*, *tidyverse*, *cluster*, *factoextra*, *gridExtra*, *wordcloud*, *tm*, *corrplot*, and *ggplot2*. The codes and data are freely available upon request.

## Results

### Description of Viral COVID-19–Related Social Media Posts on Sina Weibo and Twitter

**Figure 2:**
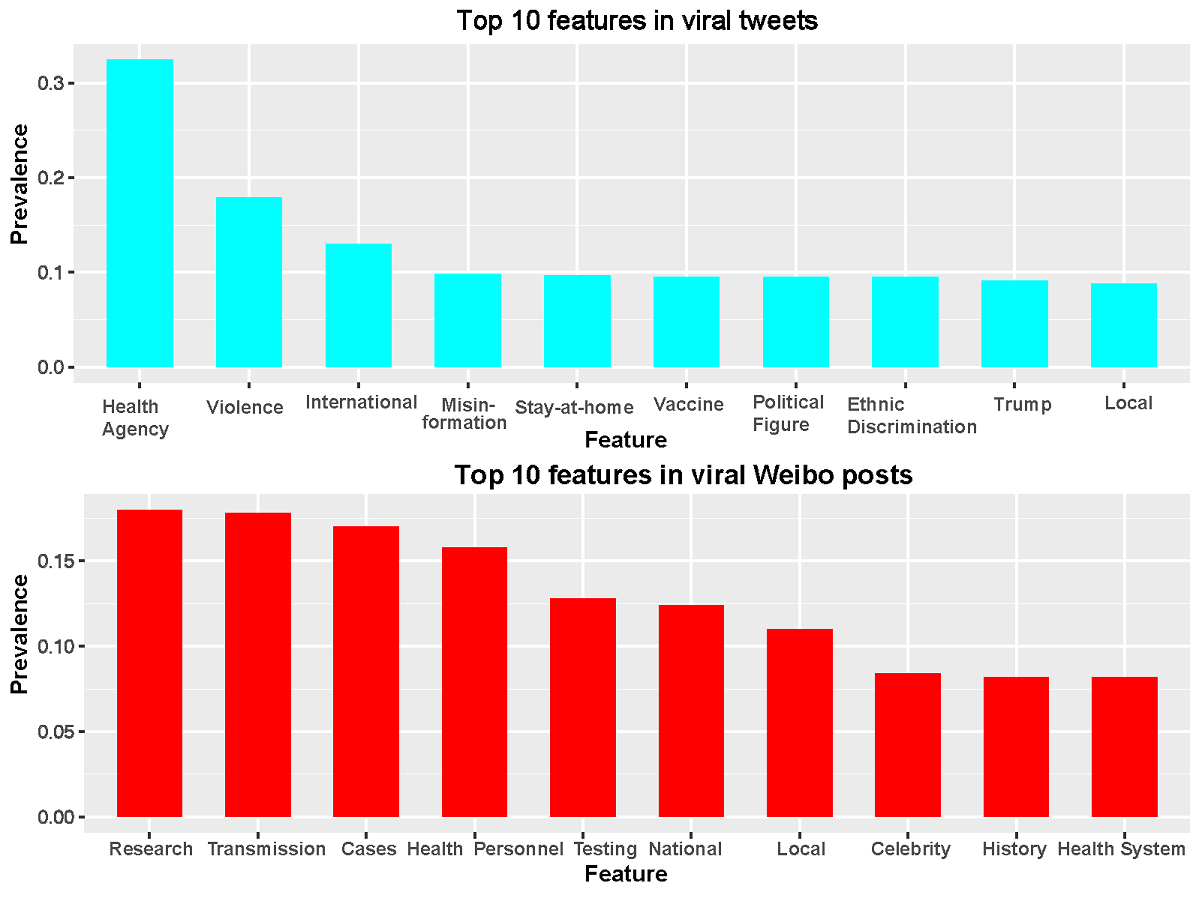
Top 10 content features and their prevalence on Twitter (top) and Weibo (bottom).

The most prevalent content features in Twitter (which has mostly Western users) were *health agency* (eg, CDC [US Centers for Disease Control and Prevention], NIH [National Institutes of Health], and WHO [World Health Organization]; 37.0%), *violence* (mostly related to domestic violence due to stay-at-home orders; 20.4%), *international relationships* (14.8%), *misinformation* (eg, mentioning *misinformation*, *disinformation*, *hoax*, *fake news*; 11.2%), *stay-at-home order* (11.0%), and *vaccine* (10.8%). The 10 most frequently mentioned content features on Twitter, along with their prevalence and ranking, are shown in Figure 2 (top panel). In general, prevalent COVID-19 content features on Twitter did not directly focus on the disease itself and the epidemic but rather on policies, politics, and other secondary societal issues, such as violence and discrimination. This finding reinforced the notion that COVID-19, like many large pandemics and emerging health issues, is not an isolated medical issue and is intertwined with complicated sociopolitical aspects. In particular, 2020 was a US presidential election year. Therefore, it was not surprising that US President Donald Trump and other former and current US office holders (eg, President Barack Obama, Vice President Joe Biden, Majority Leader Mitch McConnell, and House Speaker Nancy Pelosi) were frequently mentioned in COVID-19–related viral tweets. Given the partisan nature of the US political system, the *Republican Party* and *Democratic Party* were also consistently mentioned with COVID-19, mostly with the distinct views and countermeasures of these parties related to the pandemic. The most mentioned nonpolitician *celebrity* was Bill Gates, and mentions of his name were usually associated with content features of *vaccines* and *misinformation* (mostly vaccine-related conspiracy theories). *Discrimination* toward Chinese people, Asian Americans, and Asian people in general was also frequently mentioned. Note that these were content features and may not reflect actual discrimination and negative sentiments against these groups in the tweets. In fact, many viral tweets that mentioned *discrimination* features were advocating for the elimination of discrimination and xenophobia.

In comparison, the most prevalent content features in Weibo were *research* (18%), *transmission* (17.8%), *cases* (17%), *healthcare personnel* (15.8%), and *testing* (12.8%). The top 10 most mentioned content features on Weibo, along with their prevalence and ranking, are shown in Figure 2 (bottom panel). Compared to Twitter users, Weibo users (who are mostly Chinese) were more likely to engage in discussion of disease-related content features. Among the 10 most common content features, only *celebrity* was not directly related to the disease itself. In other words, Chinese Weibo users tended to focus on COVID-19 as a health and medical issue rather than on the associated societal and political issues discussed in Western societies. Viral Weibo posts were much more likely to mention *health personnel* and pay tribute to health care workers. *Research* on the SARS-CoV-2 pathogen and its *transmission* among human populations were also frequently mentioned, demonstrating the public interest in the state-of-the-art understanding of the emerging health crisis. Because China experienced the original 2003 severe acute respiratory syndrome (SARS) outbreak, which was caused by severe acute respiratory syndrome coronavirus 1 (SARS-CoV-1), and COVID-19 was caused by a similar coronavirus (SARS-CoV-2), the *history* of the 2003 SARS outbreak was a recurrent theme in COVID-19 Weibo posts. The *celebrities* mentioned in posts related to COVID-19 on Weibo were also very different from those on Twitter. In general, viral Weibo posts mentioned pop culture idols (eg, singers, other performing artists, and sports stars), and the sentiment was almost always positive (eg, mentions of financial, resource, and emotional support for COVID-19–impacted regions and people provided by these celebrities).

These results showed vastly different content features covered in viral posts between Weibo and Twitter, which reflected the vast differences in perception of COVID-19 in the corresponding two major sociocultural systems. In general, Twitter users (who mostly live in Western countries) were highly engaged in discussions with countermeasures, politics, and policies related to the COVID-19 pandemic. In comparison, Weibo users (mostly Chinese) tended to focus more on the disease itself, but not exclusively. Among the top 10 features, the only overlapping content feature between the two platforms was the *local* situation. Therefore, these findings reveal substantially different focuses on the COVID-19 pandemic in Chinese and Western societies, which were reflected in the most viral social media posts in cyberspace.

### Comparative Analysis of Content Features of Twitter and Sina Weibo

We further provided a quantitative comparison of content features between the two social media platforms. Out of a total of 77 content features, 3 (4%) were absent from all of the 500 most viral tweets (*comorbidity*, *eHealth*, and *suicide*), and 6 (8%) were not present in any of the 500 most viral Weibo posts (*constitution*, *curfew*, *remote working*, *major religion*, *discrimination against gender*, and *discrimination against religion*). This result also implies that viral discussions of COVID-19 on Weibo had narrower but more focused content features. There was no intersection of missing features between the two major social media platforms.

Two-sample *z* tests were used to further quantify between-platform differences for each content feature. Content features with zero prevalence (ie, never mentioned in viral social media posts on either platform) were removed to perform the *z* test correctly. Features having the most distinct prevalence between the two platforms were *health agency* (difference of prevalence [*D*]=0.25; Twitter minus Weibo; *P*<.001), *vaccine* (*D*=–0.17, *P*<.001), *shelter-in-place* (or lockdown, *D*=–0.11, *P*<.001), *cases* (*D*=0.09, *P*<.001), and *stay-at-home order* (*D*=0.10, *P*=.002). While many of these content features were among the top 20 mentioned on both social media platforms (Figure 2), we also observed that *local* situations, the only common top 10 feature in both platforms, actually had statistically significant differences (*D*=–0.11, *P*<.001). *Local* was the 6th most mentioned content feature on Weibo and the 10th on Twitter. These quantitative findings can be explained by the different sociocultural backgrounds of the users of Twitter (Western) and Weibo (Chinese).

Some features were also distributed similarly between the two social media platforms (ie, *P* values substantially greater than .05 based on the *z* test). Of them, *preparedness* (*D*<0.01, *P*=.90), *discrimination against ethnicity* (*D*<0.01, *P*=.96), *prevention* (*D*<0.01, *P*=.97), *recovery* (*D*<0.01, *P*=.97), *ecosystem* (*D*<0.01, *P*=.97), *masks* (*D*<0.01, *P*>.99), and *Trump* (*D*<0.01, *P*>.99) were the least distinct features. These features represent the common ground regarding COVID-19 between the two social media platforms and the two underlying sociocultural systems.

The missing content features revealed a discrepancy between viral and nonviral discussions of COVID-19 on social media. As mentioned earlier, the comprehensive content feature coding scheme was originally developed from a random sample of posts, most of which were nonviral posts with <5 reposts. We speculated that certain controversial content features (especially those related to policy and politics on Twitter) facilitated the spread of certain posts on social media and caused them to go viral. Posts that are less controversial typically do not gain much attention and do not go viral on social media. However, we must point out that content features are only one reason that a post can go viral. Other aspects include temporality (ie, when the post was published relative to the epidemic), property of the original posting user (eg, number of followers), and the severity of the pandemic at that time and place.

**Figure 3:**
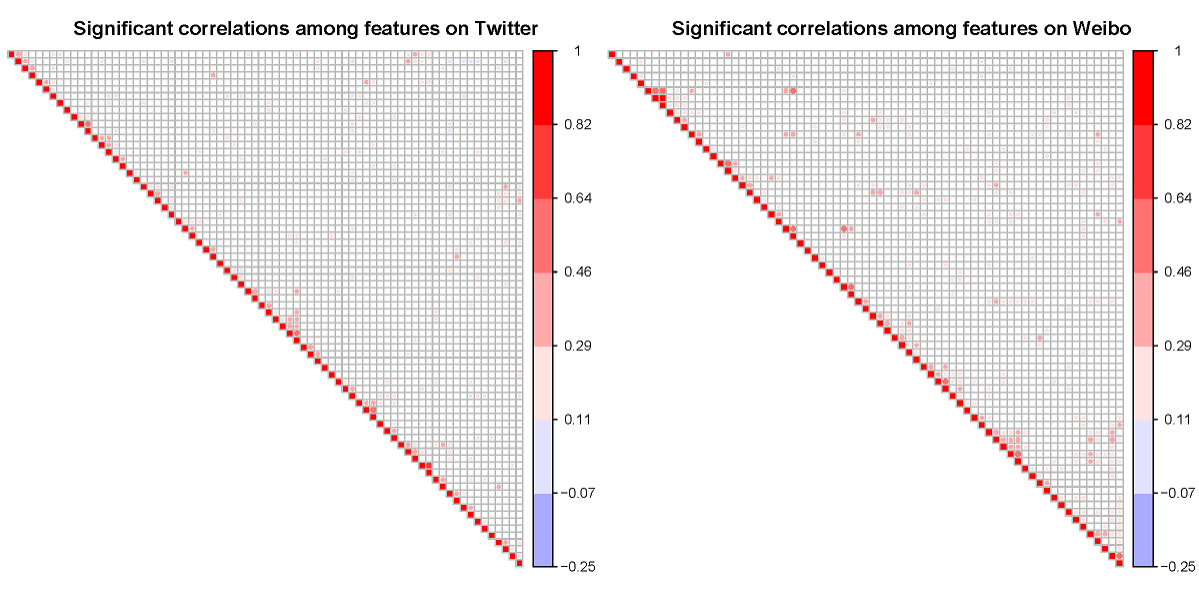
Significant Pearson correlations (*P*<.05) among content features on Twitter (left) and Weibo (right). The complete list of the 77 content features on the x- and y-axes can be found in Multimedia Appendix 1.

Significant Pearson correlations (*P*<.05) are shown in Figure 3 for Twitter (left) and Weibo (right) posts, respectively. In general, significantly correlated content feature pairs were more abundant on Weibo than on Twitter. One possible explanation is that Twitter has a 280-character length limit for posts. Therefore, content features in each tweet were limited, and concurrent content features in the same tweet were less frequent. On the other hand, Sina Weibo allows up to 2000 characters; therefore, it is possible to include much more content in a Weibo post than in a tweet. Consequently, a Weibo post can accommodate more content features than a tweet. Viral COVID-19 tweets included an average of 2.37 content features, and viral Weibo posts contained 2.78 content features. However, most viral Weibo posts used URLs to pack in more information and keep the post concise rather than including everything in the main post content. Therefore, the 2000 character limit is only a theoretical upper limit and was rarely reached, especially for viral Weibo posts.

Note that Weibo is subject to censorship toward certain content features. For example, although US President *Trump* was mentioned quite a few times in viral Weibo posts, President Xi of China is not an allowed topic on Weibo and Chinese cyberspace in general. Therefore, there was no equivalent content feature to *Trump* on Weibo. Other *political figures* in China, such as the governor of Hubei (Yong Ying), are generally permitted by censors to be mentioned and commented on in Weibo posts.

### Dimension Reduction and Clustering Analysis of Content Features

**Figure 4:**
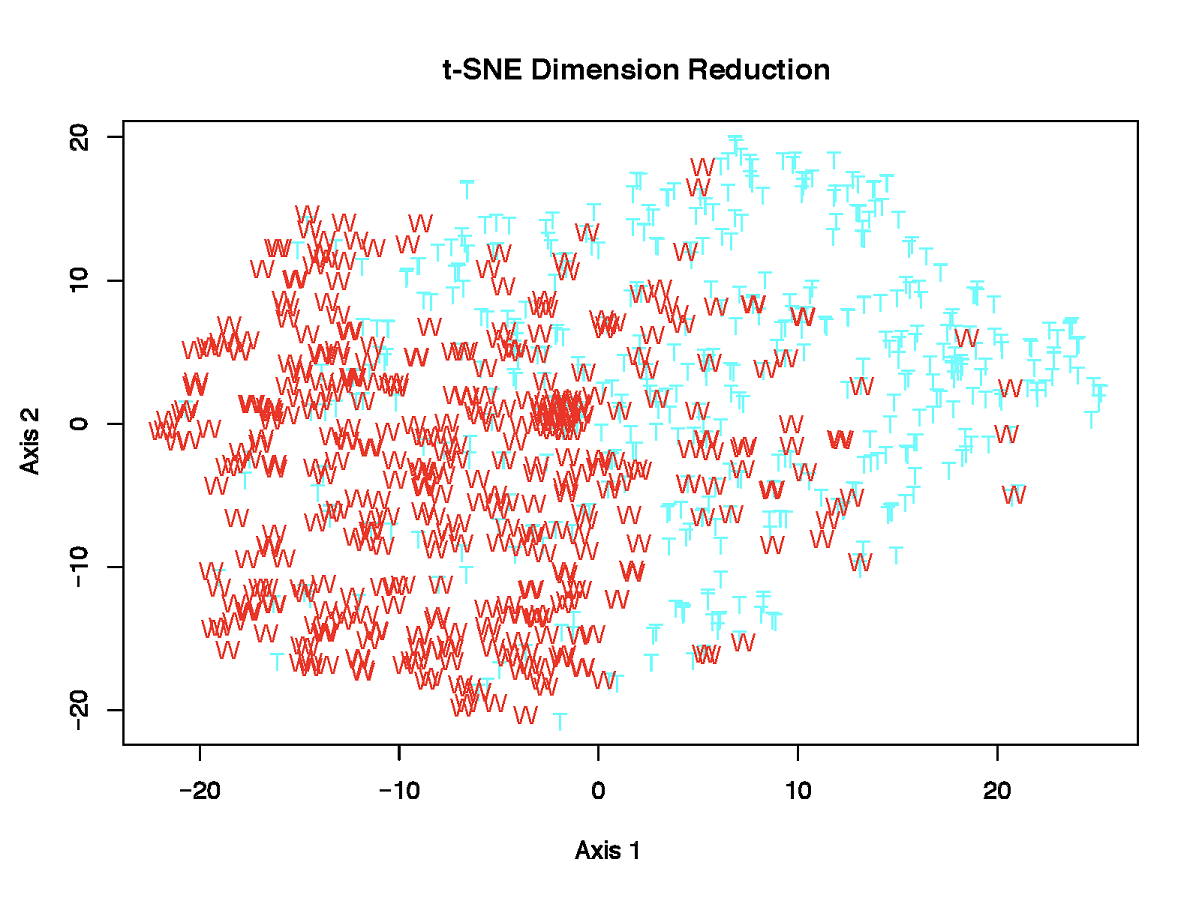
*t*-SNE results for viral COVID-19 tweets (T) and Weibo posts (W).

The machine learning dimension reduction *t*-SNE results for Twitter and Weibo are shown in Figure 4. These figures show how content features are distributed and associated in the reduced 2D space instead of the original 77-dimensional feature space. It is very clear that the content features have distinct distribution patterns between the two social media platforms in the reduced 2D space. This reinforces our previous findings on the variability of content features across the sociocultural spectrum.

**Figure 5:**
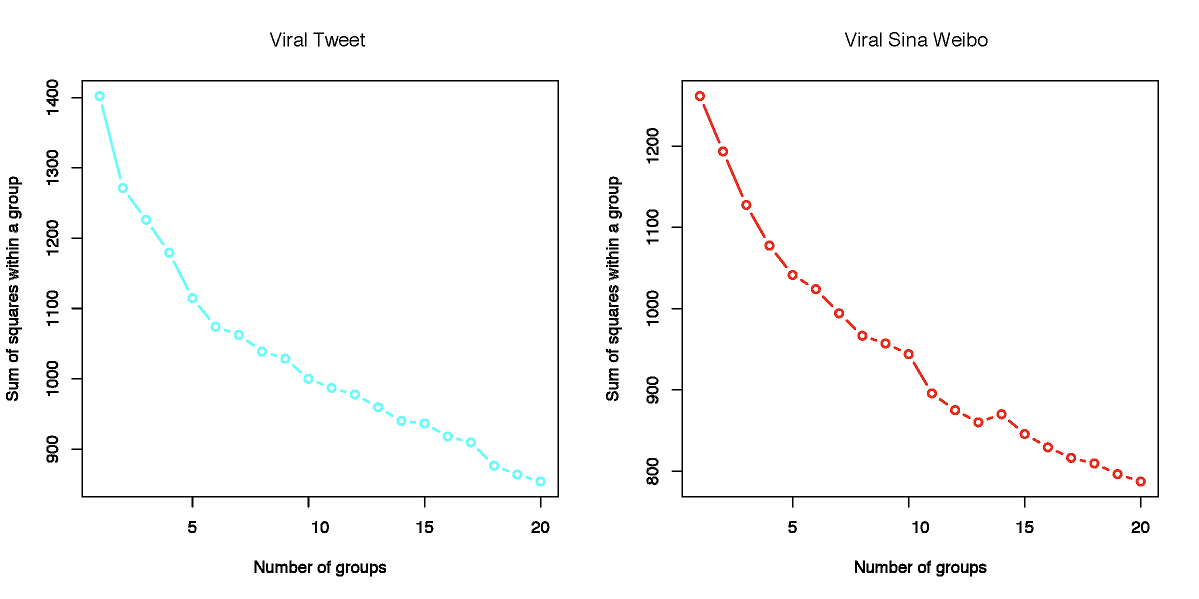
Numbers of clusters and within-group sums of squares for tweets (left) and Weibo posts (right).

The number of optimal clusters on Twitter (*k*_t_) was determined as 6 from Figure 5 (left), while the number of optimal clusters (*k*_s_) on Weibo was found to be 5 from Figure 5 (right). Therefore, not only were content features regarding COVID-19 distributed differently between the two social media platforms, but their associations (eg, clusters) within posts were also distinct between the two platforms. Note that these clusters were identified by the data-driven unsupervised machine learning technique, and these clusters did not necessarily align with the 6 manually developed major categories.

**Figure 6:**
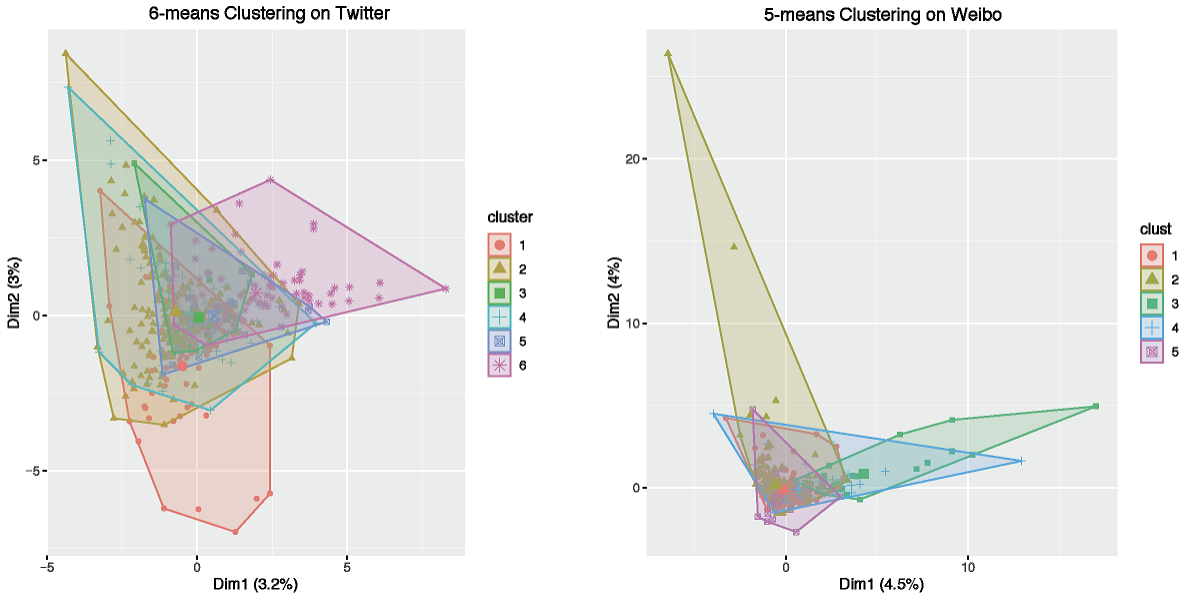
*k*-means clustering on viral tweets (left) and Weibo posts (right).

We further show the *k*-means clustering results of the content features on Twitter and Weibo in Figure 6 (left and right, respectively). The clustering patterns were substantially different between the two social media platforms. The sizes of the 6 distinct clusters on Twitter were 154, 107, 96, 62, 42, and 39. The total sum of squares (TSS) across all 6 clusters was 1402. The total within-cluster sum of squares (TWSS) was 1079, and the total between-cluster sum of squares (TBSS) was 323 on Twitter. Note that TSS = TWSS + TBSS. In comparison, the 5 cluster sizes of Weibo posts were 218, 106, 81, 67, and 28. The TSS, TWSS, and TBSS on Weibo were 1262, 1034, and 228, respectively. Therefore, all sums of squares were much smaller on Weibo than on Twitter. In addition, the two dimensions (the x- and y-axes in Figure 6) were also much smaller on Twitter (3.2% and 3%) than on Weibo (4.5% and 4%). All these results reveal that COVID-19 content features in viral Weibo posts were more similar across different posts than those in Twitter posts. Twitter showed a more diverse array of content features among different tweets.

## Discussion

### Theoretical Innovation

This study is the first of its kind to comprehensively characterize the content features of discussions regarding a large pandemic on social media across the sociocultural spectrum. We showed the vast differences in topic content features of viral social media posts in Twitter and Weibo, the two most influential social media platforms in China and the West during the COVID-19 pandemic. In general, viral social media posts in China focused on cases and prevention, which are topics that are more related to COVID-19 as a health issue. However, as a comparison, most viral tweets regarding COVID-19 focused more on policies and politics, including *stay-at-home* orders, President *Trump*, and other *political figures*. Through various analytical methods, social media data provided a new angle to explore and understand public discourse of the COVID-19 pandemic and associated social, political, and economic issues. Details of these discussions in virtual cyberspace may provide insights on the actual disease epidemic in the real world. For example, analyzing public perception of various NPIs, such as *social distancing*, mandatory *mask-wearing*, and *stay-at-home* orders, would provide an estimation of the compliance with these NPIs, which determine the case counts and epidemic trajectory in a region. This concept echoes the original idea of infodemiology, which uses a time series of social media post counts related to a health issue (eg, COVID-19) as an indicator of actual case counts [14-16]. In addition to the number of social media posts, we will be able to further extract fine-grained perceptions of the risk and NPIs of COVID-19 and extend the application of infodemiology.

### Technical Advances

To achieve an effective comparison across the sociocultural spectrum regarding the COVID-19 pandemic on social media, we developed a comprehensive content analytical workflow. This analytical workflow was specifically designed for transboundary infectious diseases (eg, outbreaks and pandemics of infectious diseases) that have complicated sociocultural contexts. Compared to the commonly used LIWC [31], our workflow, especially the coding scheme, has several advantages. First, LIWC is a general content analytical tool that ignores many important content features during the COVID-19 pandemic. Our coding scheme is tailored to the complicated and interacting health, social, cultural, and political nuances of transboundary infectious diseases. Therefore, our coding scheme is able to capture a much more comprehensive and detailed content features in web-based discussions regarding transboundary infectious diseases. Second, LIWC uses proprietary algorithms to calculate individual scores of different features, and the exact interpretation of the numeric values is not readily comprehensible. In contrast, our coding scheme is 0-1 binary, where 1 indicates that the content has a feature and 0 indicates that it does not. This coding is clearer than the obscure LIWC scores. In addition, LIWC scores vary substantially (from 0 to 100) among different features. Certain features that have large values in LIWC tend to dominate and overshadow other features; thus, further analysis is prone to bias. Our coding scheme is consistent, as all features have the same coding scheme. Finally, LIWC is difficult to directly apply to non–Indo-European languages; therefore, direct comparison between sociocultural contexts with LIWC is almost impossible. In contrast, our coding scheme is context-free and can be applied to virtually any language and any region. The coding scheme itself is also flexible. Researchers can easily add and modify content features as necessary when working with other health issues beyond COVID-19. The coding scheme can be retrofitted to understand communications on previous events (eg, the 2016 Zika event). We can easily add, remove, or revise corresponding content features related to the specific health issues we are exploring.

### Limitations of the Current Study and Future Directions

This study adopts a static view of all viral social media posts for comparative analysis between two sides of the sociocultural spectrum in a given period of time. However, for a large and ongoing pandemic, time is another major influential factor that is associated with the actual progress of the pandemic. Our previous studies showed that the Zika case series was strongly associated with the Zika discussion trend on Twitter in 2016 [24,25]. Similarly, future studies can be expended to explicitly characterize how various content features evolve with time in different regions. The ongoing COVID-19 pandemic case series can be predicted by certain content features (eg, regarding NPIs), similar to the previously discussed infodemiology approach.

We used the number of reposts (ie, retweets or shares) as the definition of a viral social media post. One limitation is that we did not consider the possibility of automatic reposting by bots or cyborgs. Therefore, it is possible that the large number of reposts may not accurately represent and reflect the public perception of an issue. Bots and cyborgs, however, are not necessarily associated with misinformation. Bots and cyborgs can be used as tools to quickly disseminate information on social media platforms for other reasons, such as advertising. A future direction of this study is to identify other definitions of viral posts (eg, posts with a large number of likes, favorites, or comments).

Viral social media posts are only one of many attributes of social media discussion. Our initial assessment showed that >75% of tweets and >80% of Weibo posts regarding COVID-19 did not receive any attention on social media. This number is similar to our previous finding that 76% of all Zika-related tweets were never retweeted [25].

To characterize web-based public discourse related to COVID-19 and other emerging health issues accurately and comprehensively, we will continue studying these nonviral social media posts on different platforms. However, given the ever-increasing volume of social media posts, effective sampling strategies are a priority. Effective sampling is a necessity to provide a less biased depiction of content features. Data mining of nonviral posts regarding COVID-19, especially on sentiment toward NPIs, will provide a more accurate estimation of compliance with NPIs in different regions at different stages of the pandemic. We will also be able to further compare and contrast how the distributions of content features differ between viral and nonviral post groups as well as across the sociocultural spectrum.

In this study, we depict how NPIs of COVID-19 have been mentioned on social media across the sociocultural spectrum. Because this study focuses on providing a neutral and objective characterization of content features in COVID-19–related discussions, it does not consider subjective sentiment toward specific NPIs. However, individual and societal perception toward NPIs can be strong influencing factors during the COVID-19 pandemic. For instance, positive sentiment toward *mask-wearing* and *social distancing* may reflect actual compliance with these NPIs in society and hence help reduce the risk of transmission. On the other hand, negative sentiment toward these NPIs may lead to noncompliance and facilitate COVID-19 transmission in the real world. In a future study, we will further integrate objective content features and corresponding sentiment and/or emotion to provide a more comprehensive understanding of public perceptions.

Finally, this study relies on human coding of content features, which is substantially labor-intensive. For instance, adequate and proper training is required to achieve high intercoder reliability before each coder can perform independently. In comparison, the LIWC algorithm is automated and relatively easy to use. We are still at the early development stage of a novel analytical workflow that is similar to LIWC. We expect to develop at least a semiautomated and semisupervised machine learning method for quick and effective web-based health information processing and annotation. To achieve this ambitious goal, we envision a crowd-sourcing approach that will enable ardent citizen scientists and volunteers worldwide to help further manually code more social media posts, create an even larger corpus, and develop state-of-the-art semisupervised or supervised machine learning pipelines to automate the process. The eventual product will be able to automatically extract content features from social media posts regarding health issues and can further guide effective health communications during emergencies.

## Appendix

Multimedia Appendix 1 Coding book of COVID-19 content features.

## Abbreviations

CDC: US Centers for Disease Control and Prevention
*D*: difference of prevalence
LIWC: linguistic inquiry and word count
NIH: National Institutes of Health
NLP: natural language processing
NPI: nonpharmaceutical interventions
PHEIC: public health emergency of international concern
SARS: severe acute respiratory syndrome
SARS-CoV-1: severe acute respiratory syndrome coronavirus 1
*t*-SNE: t distribution stochastic neighbor embedding
TBSS: total between-cluster sum of squares
TSS: total sum of squares
TWSS: total within sum of squares
WHO: World Health Organization
