# Supplementary material for: A Novel Machine Learning Framework for Comparison of Viral COVID-19–Related Sina Weibo and Twitter Posts: Workflow Development and Content Analysis
Source: J Med Internet Res. 2021 Jan 6;23(1):e24889. doi: 10.2196/24889 (PMC7790734; doi:10.2196/24889)
Supplement: Multimedia Appendix 1 [file jmir_v23i1e24889_app1.pdf]

Codebook for Evidence-based COVID-19-Related Tweets and Sina Weibo

Please note that we do not consider whether the information is true or not when coding the list of features (bullet points). For each tweet, your task is to choose all the features that are present based on your interpretation of the tweet content.

This document provides the list of features and their detailed descriptions. Please note that the features are organized by group (both highlighted in bold and underlined).

- You should record each of your selected features from the dropdown menu in the corresponding cell. You are allowed to choose more than one feature under each category by recording them in different columns.
- You also have the option of suggesting new features as appropriate by inserting them into Remark (last) column.
- You can simply leave a cell blank if none of the features of a main category is present in the tweet.

Clinical and Epidemiology

"Clinical" means interaction with physicians for diagnosis and treatment. Epidemiology is at population-level. In this study it is not necessary to separate them apart.

- Mentioned any **symptom(s)/sign(s)** associated with COVID-19 (the list of symptoms can be found on CDC's website https://www.cdc.gov/coronavirus/2019-ncov/symptoms-testing/symptoms.html):
    -
- Mentioned anything related to **transmission** of COVID-19 (such as any mode of transmission regardless of reliability; through aerosol, through airborne, through food, through contaminated surfaces, through mucus, etc.) Note: if mentioned transmission from pet animals but not their role as companion, then code as transmission without companion category. Otherwise, code BOTH transmission AND companion animals.
    -
- Mentioned anything related to **diagnoses or testing** of COVID-19 (such as qRT-PCR, swab, testing facility, testing experience, etc.)
    -
- Mentioned anything related to **treatment** of COVID-19 (such as any fake treatments, anything that can treat COVID-19 (true or not), statements that there is no specific medicine or vaccine for COVID-19 virus, there is only ways to treat the symptoms, getting plenty of rest, drinking fluids to prevent dehydration, taking medicine such as acetaminophen (Tylenol) to reduce fever and pain, avoiding taking aspirin, hydro-

cloriquine, and lysol injection). Note: cure may be labeled as treatment/treating. It is the clinical consequence (prognosis) of treatment.

- Mentioned anything related to **prevention** of COVID-19 (such as wearing masks, washing hands, keeping social distance, stay-at-home order, etc. Note someone might confuse prevention with treatment.)
- Mentioned **vaccine (or vaccination, immunization)**. Note: technically, vaccination is a type of prevention, but since we have not yet had a COVID-19 vaccine, we treat it as a separate content feature, and may not always concur with prevention.
- Mentioned anything related to the number of **cases** of COVID-19 (total cases and/or new cases per day). Note: must describe the number(s) explicitly.
- Mentioned **history** of COVID-19 and SARS-CoV-2 (and related virus such as SARS-CoV, MERS, etc.):
- Mentioned **recovery** from COVID-19
- Mentioned **clinical consequences** of COVID-19 (e.g., ARDS, death, etc.)
- Mentioned **risk factors** of COVID-19 (e.g., age, ethnicity, etc.)
- Mentioned **comorbidities** of COVID-19 (Comorbidity means concurrent diseases, mostly chronic diseases, e.g., CVD, diabetes, COPD, etc.) Note: many comorbidities are also risk factors of COVID-19, but not always.
- Mentioned **pharmacy** (e.g., prescription, Rx, etc.)
- Mentioned **eHealth or mHealth** (e.g., tele-diagnosis, online consulting, p2p health forum, etc.):

- Mentioned anything about the **healthcare system** (e.g., hospital, clinic, nursing home, etc.)
    -

- Mentioned anything about **healthcare personnel** (e.g., clinician, nurse, etc.)
    -

<u>**Countermeasures and COVID-19 Related Resources**</u>

Countermeasures are defined as anything that can reduce the transmission of COVID-19 at both personal and population levels. Resources are materials and/or assets that individuals can adopt or reference in understanding or overcoming adverse situations like COVID-19.

- Mentioned **masks** (regardless of attitude):
    -

- Mentioned **other medical supplies** (e.g., ventilator, ECMO, gloves, other PPE):
    -

- Mentioned **disinfection** (e.g., spray, etc. Note that disinfection may be a sub-category of prevention):
    -

- Mentioned **food** (e.g., fruit, meat, herb, etc.)
    -

- Mentioned **exposure** to COVID-19 (e.g., in close contact with patients)
    -

- Mentioned anything related to **contact tracing** of COVID-19 (such as using apps, questionnaire, etc.)
    -

- Mentioned **technology** (e.g., AI, ML, may not directly related to COVID-19)
    -

- Mentioned anything about **research** (e.g., experts, research on understanding various aspects of COVID, etc)
    -

- Mentioned **online resources** of COVID-19 (regardless of credibility/reliability)
    -

- Mentioned **companion animals, emotional support animals (ESA)** of COVID-19 (including but not limited to domestic dogs, cats, horses, rabbits, etc.) Note: code

companion animals only when the animals are considered as companions, NOT wild animals such as bats.

  -

<u>Policies and Politics</u>

Policies are developed by governmental agencies and health organizations, and are sometimes enforced by the government. Politics are tuned towards the U.S. political system with federal and state/local government; three components in federal government (legislative, administrative, and judicial); partisan politics (GOP and Democratic), etc.

- Mentioned **social distancing**:
  -
- Mentioned **stay-at-home order**:
  -
- Mentioned **shelter-in-place**:
  -
- Mentioned American **constitution**:
  -
- Mentioned **laws and/or judicial system**:
  -
- Associated COVID-19 and its outbreak with **politics** and 2020 Presidential **election**:
  -
- Associated COVID-19 and its outbreak with **GOP**:
  -
- Associated COVID-19 and its outbreak with **Democratic Party**:
  -
- Associated COVID-19 and its outbreak with **President Trump** (must explicitly mention Trump, POTUS, President, etc.)
  -
- Associated COVID-19 and its outbreak with **Political figures** (excluding Trump)
  -
- Associated COVID-19 and its outbreak with **legislation** (e.g., congress, senate)
  -

- Mentioned anything related to **economic policy** related to COVID-19 (e.g., government actions that is intended to influence or control the behavior of the economy, including setting taxation, government budgets, money supply, interest rates, and labor market, and national ownership, e.g., economic relief to assist workers and small businesses)
    -

- Mentioned **curfew**:
    -

- Mentioned public sector (e.g., health agencies such as WHO, CDC, NIH, etc.) **responses**/**reactions** towards the COVID-19 and its outbreak:
    -

- Mentioned **federal** government (e.g., central government in which states form a unity but remain independent in internal affairs)

<u>Public Responses and Societal Impact</u>

Public responses are actions taken to respond to COVID-19 at both individual and societal levels. Societal impacts refer to the direct and indirect impacts of COVID-19 on various areas and levels of the society (e.g., interpersonal relationship, working, education, traveling, etc).

- Mentioned **preparedness** (e.g., stocking supplies):
    -

- Mentioned **shortage** of supplies due to COVID-19 (e.g., toilet paper, sanitizer)
    -

- Mentioned **financial impact** of COVID-19 and its outbreak on the financial well being of individuals/families/community (such as on unemployment, access to service, wealth, etc.):
    -

- Mentioned **interpersonal relationship** (e.g., family, co-workers, friends, etc.) Note: depending on its prevalence, may consider developing more specific relationship content features.
    -

- Mentioned **riot/unrest**:
    -

- Mentioned **protest** (regardless of reason):
    -

- Mentioned **non-international traveling** (e.g., driving within or to another state within the nation):
    -

- Mentioned **international traveling** (e.g., international flight):
    -

- Mentioned anything about **college education** (e.g., university closing, transition to online, etc)
    -

- Mentioned anything about **non-college education** (e.g., K, pre-K, kindergarten, daycare, etc)
    -

- Mentioned **remoting working**
    -

- Mentioned **business** (an organization or enterprising entity engaged in commercial, industrial, or professional activities, e.g., restaurants, tourism, sports, retail, etc.)
    -

- Mentioned **sports and activity** (e.g.,indoor or outdoor activities)
    -

- Mentioned **mental health (**e.g., depression, anxiety**)**
    -

- Mentioned **suicide** (ideation and/or activity)
    -

- Mentioned general public (including individual) **responses/reactions** towards the COVID-19 and its outbreak:
    -

- Mentioned events not directly related (**unrelated**) to COVID-19 (e.g., pearl harbor attack; may confound with politics and government response):
    -

- Mentioned **mainstream religion (**e.g., the big three, Buddhism, Judaism, etc**)**
    -

- Mentioned **folk religion** (or alternative healing, spirit healing, etc)
    -

- Mentioned **celebrity** (non-political):
    -

- Mentioned **product promotion** (e.g., advertising)
    -

- Mentioned **ecosystem/ecosphere** (e.g., bracing the nature, etc.)
    -

Spatial Scales

Spatial scale is the extent of an area at which COVID-19 occurs, ranging from local, state, national, and international levels.

- Mentioned anything **local** (e.g., about home town, city)
    -

- Mentioned anything **state** (e.g., specific state in the U.S. or specific province in China, etc.)
    -

- Mentioned anything at **national**
    -

- Mentioned anything **international** (e.g., cases in other countries, or international collaboration/hostility)
    -

Social Problems

A social problem is any condition or behavior that has negative consequences for large numbers of people because of COVID-19 and that is generally recognized as a condition or behavior that needs to be addressed. It is a common problem that many people strive to solve, and often the consequence of factors extending beyond an individual's control.

- Mentioned **discrimination towards certain countries**. Note: must explicitly specify the country or countries.
    -

- Mentioned **discrimination towards certain regions** (within a country).
    -

- Mentioned **discrimination towards certain ethnicity groups**
    -

- Mentioned **discrimination towards certain professions** (e.g., healthcare professionals)
    -

- Mentioned **discrimination towards certain gender/sex orientation**
    -

- Mentioned **discrimination towards certain age/age groups**
    -

- Mentioned **discrimination towards certain religion**
    -

- Mentioned **discrimination towards certain food preference** (e.g., carnivorous, vegetarian, etc.)
    -

- Mentioned **violence** (towards others). Note: different from mentioning riots. Violence must have a specific target (e.g., any of the aforementioned groups or family member).
    -

- Mentioned **profanity, and made derogative, offensive, and uncivil comments** (e.g., WTF, damn, name-calling, etc.)
    -

- Mentioned **misinformation** or similar terms (e.g., fake news, disinformation, etc.)

<u>Other consideration</u>

- Perceived **Sentiment** (e.g., positive, neutral, negative). Note: This is not binary
    - 0 = negative, 1 = neutral, 2 = positive

- Perceived **Emotion**(s) (e.g., anger, sad, happy, fear, surprise, disgust). Note: This is not binary
    - 9=trust, 8=distrust, 7=surprise, 6= sarcasm, 5 = anger, 4 = sadness, 3 = fear, 2= disgust, 1 = happiness
